# Comparative Proteome Analysis of hAT-MSCs Isolated from Chronic Renal Failure Patients with Differences in Their Bone Turnover Status

**DOI:** 10.1371/journal.pone.0142934

**Published:** 2015-11-17

**Authors:** Murat Kasap, Itır Yeğenağa, Gurler Akpinar, Mehmet Tuncay, Ayça Aksoy, Erdal Karaoz

**Affiliations:** 1 Department of Medical Biology/DEKART Proteomics Laboratory, Kocaeli University Medical School, Kocaeli, Turkey; 2 Department of Internal Medicine, Nephrology, Kocaeli University Medical School, Kocaeli, Turkey; 3 Department of Stem Cell, Center for Stem Cell and Gene Therapies Research and Practice, Institute of Health Sciences, Kocaeli University, Kocaeli, Turkey; 4 Liv Hospital Center for Regenerative Medicine and Stem Cell Research and Manufacturing, İstanbul, Turkey; Second University of Naples, ITALY

## Abstract

The relationship between the stem cells and the bone turnover in uremic bone disease due to chronic renal failure (CRF) is not described. The aim of this study was to investigate the effect of bone turnover status on stem cell properties. To search for the presence of such link and shed some light on stem-cell relevant mechanisms of bone turnover, we carried out a study with mesenchymal stem cells. Tissue biopsies were taken from the abdominal subcutaneous adipose tissue of a CRF patient with secondary hyperparathyroidism with the high turnover bone disease. This patient underwent parathyroidectomy operation (PTX) and another sample was taken from this patient after PTX. A CRF patient with adynamic bone disease with low turnover and a healthy control were also included. Mesenchymal stem cells isolated from the subjects were analyzed using proteomic and molecular approaches. Except ALP activity, the bone turnover status did not affect common stem cell properties. However, detailed proteome analysis revealed the presence of regulated protein spots. A total of 32 protein spots were identified following 2D gel electrophoresis and MALDI-TOF/TOF analyzes. The identified proteins were classified into seven distinct groups and their potential relationship to bone turnover were discussed. Distinct protein expression patterns emerged in relation to the bone turnover status indicate a possible link between the stem cells and bone turnover in uremic bone disease due to CRF.

## Introduction

Uremic bone disease (UBD) is a destructive complication of CRF. UBD has recently been redefined as the bone and mineral disease in CRF [1]. Severity of UBD affects the rate of morbidity, mortality and quality of life in CRF patients. UBD appears mostly in complicated clinical presentations. While some patients suffer from secondary hyperthyroidism (SHPT) (High Turnover Bone Disease, HTBD) and associated bone abnormalities, others might have adynamic bone disease (ADBD) (Low Turnover Bone Disease-LTBD) and osteoporosis [1–3]. Furthermore, extra osseous calcification occurs often in CRF patients with LTBD [4–6]. Some etiologic factors affecting the type of bone disorder development in CRF were reported recently, but there are some contradictions in this field [2]. There's still a need for understanding the molecular basis that will help understanding of the etiology of the type of bone disorders in CRF.

Stem cells are undifferentiated cells that can divide, differentiate and self-renew to produce new stem cells in multicellular organisms [7]. They can be used in biomedical research, drug discovery, and toxicity testing, as a model in understanding diseases and most importantly for therapeutic purposes in regenerative medicine [8, 9]. It was demonstrated that systemic application of adipose-tissue-derived stem cells induced expression of the proteins to stimulate osteoblastic and osteoclastic functions in C57BL/6J mice and treated osteoporosis [10]. Adipose tissue derived stem cells (hAT-MSCs) is one of the most practical postnatal stem cells to use in research because of the ease to obtain without any ethical concern. In addition to these aspects, hAT- MSCs may be preferred in bone disease research due to several other reasons; 1) In hypercalcemic status, adipose tissue may get calcified [11–14], 2) During the follow up of CRF patients, soft tissue calcifications occur [4], 3) Adipose tissue is an easily accessible tissue and may be used to study molecular mechanisms of bone diseases [11].

The hypothesis of this study was based on the possibility that adipose tissue calcification in CRF patients might originate from differentiation of the stem cells present in the adipose tissue. If the stem cells isolated from adipose tissue of different degrees of parathormon function were studied by proteomic analysis; then some important clues about the molecular mechanisms behind the soft tissue calcification may be obtained. Therefore, we performed a study in which hAT-MSCs isolated from two patients with different types of bone status, namely HTBD with CRF and LTBD with CRF were compared both at the cellular and molecular levels. Furthermore, the patient with HTBD underwent PTX surgery, which allowed us to make further comparisons of hAT-MSCs isolated before and after operation. hAT-MSCs from a healthy control were also included for comparisons.

The method of choice in this study was 2DE-based proteomics, because investigation of changes in global proteome level may provide significant information about changes at the protein level, post translational modifications (PTMs) and molecular pathways [15]. Comparison of the protein expression profiles of stem cells isolated from hAT revealed the presence of a conserved protein pattern that included 70 spots, of which 32 were reliably identified by MALDI-TOF/TOF and shown to be regulated. In addition, the cells were compared on the basis of cellular morphology, expression of common stem cell markers and osteoblastic differentiation potentials as well as their ALP and telomerase activities. This is the first study presenting a list of proteins that may have importance in understanding of the differences between HTBD and LTBD in CRF.

## Material and Methods

This study was approved by Ethical Committee of Kocaeli University. Written informed consent from the donor or the next of kin was obtained to use these samples in research.

### Isolation and culture of hAT-MSCs

Tissue biopsies were taken from the abdominal subcutaneous adipose tissue of the following individuals: (1) a CRF patient with HTBD. This patient underwent PTX and one week later another sample was taken from this patient after PTX (2) a CRF patient with LTBD (3) a healthy control (Table 1).

**Table 1:** Clinical and main laboratory findings of the patients.

Name/age/gender	Clinical condition	Bone status	PTH*(pg/mL)	Ca(mg/dL)	P(mg/dL)	ALP**(U/L)
**MY/50/M**	CRF***-Pre-PTX****	High turnover	648	10.6	2.1	186.1
**MY**	CRF-Post-PTX	Low-PTH turnover	0	7.7	4.2	86
**SO/70/M**	CRF-ADBD	Low-turnover	70.2	8.6	2.6	103

*Parathormon.

**Alkaline phosphatase.

***Chronic renal failure.

****Parathyroidectomi operation.

Tissue samples were washed several times with Hanks’ balanced salt solution (HBSS) with 5% antibiocantimycotic solution without calcium and magnesium to remove blood (LifeTech, USA). A single cell suspension of adipose tissue cells was obtained by using enzymatic digestion and mechanical means. Hat-MSCs were then isolated as described by Ertas, G. et al. [16]. After cells reached 80–90% confluency, they were treated with 0.025% trypsin-EDTA for 3 min. The released cells were collected, centrifuged, and replaced at a rate of 1:3 and 1:4 for subculture.

### Characterization of MSCs by Flow Cytometry

A FACS Calibur (BD Biosciences, San Diego, USA) flow cytometry instrument was used in characterization experiments. The forward and side scatter profiles gated out debris and dead cells were analyzed using Cell Quest software (BD Biosciences). Antibodies (Becton Dickinson) against CD3, CD4, CD5, CD7, CD8, CD10, CD11b, CD13, CD14, CD15, CD17, CD19, CD29, CD33, CD34, CD44, CD45, CD71, CD73, CD90, CD106, CD123, CD133, CD138, CD146, CD166, HLA-ABC, HLA-G and HLA-DR were used for immunophenotyping of MSCs [17].

### In vitro adipogenic differentiation

Cells from passage three (0,35x10^5^ cells/cm^2^) were seeded onto coated type I collagen coverslips in 6-well plates for adipogenic differentiation. The adipogenic medium, LG-DMEM, contained 10% FBS, 0.5 mM isobutyl-methylxanthine (IBMX-Sigma-Aldrich), 10^−6^ M dexamethasone (Sigma-Aldrich, Fluka Chemie AG, Buchs, Switzerland), 10μg/ml insulin (Invitrogen/GIBCO), 200M indomethacin (Sigma-Aldrich), and 1% penicilin/streptomycin (Invitrogen/GIBCO). The differentiation lasted for four weeks and the medium was replaced twice a week. Intracellular lipid droplets, an indicator of the adipogenic differentiation were confirmed by Oil Red O staining.

### In vitro osteoblastic differentiation

For osteoblastic differentiation, cells from passage three (0,3x10^5^ cells/cm^2^) was grown in LG-DMEM supplemented with 100 nM dexamethasone (Sigma-Aldrich), 0.05M ascorbate-2-phosphate (Wako Chemicals), 10 mM β-glycerophosphate (Sigma-Aldrich), 1% penicilin/streptomycin and 10% FBS. The differentiation was accomplished within four weeks and the medium was replaced twice a week during the incubation. Alizarin Red staining was performed to demonstrate that osteoblastic differentiation was achieved.

### Alizarin red S staining

For staining, the cells were fixed for 5 min in ice-cold 70% ethanol and then allowed to dry. Cells were stained with 2% alizarin red S (pH 4.1–4.3) solution for 30 s to 1 min, then washed with distilled water (20 dips). The stained cells were dehydrated in acetone (20 dips), fixed in acetone-xylene (1:1) solution (20 dips), cleared with xylene (20 dips), dried and mounted in mounting medium.

### ALP activity

ALP activity assay was performed at days 1, 4, 7, 14, and 21. Cells were washed with PBS for three times and protein extraction was performed using Mammalian Protein Extract Reagent (M-PER) (Thermo Scientific). p-Nitrophenyl Phosphate (Sigma Aldrich, N7653) was used as the liquid substrate to detect ALP activity and the incubation of the cells with the liquid substrate lasted for 30 min. A VersaMax Microplate Reader (Molecular Devices) was used to measure the optical density at 405 nm. Bicinchoninic Acid Protein Determination method (SigmaAldrich) was used to measure total protein concentration of the cell lysate. ALP activity was expressed as OD 405 nm/mg of protein. Three technical replicates of each sample were performed.

### Telomerase activity

Telomerase activity was determined by the conventional telomeric repeat amplification protocol (TRAP). TRAP TeloTAGGG PCR enzyme-linked immunosorbent assay (ELISA) kit (Roche, Mannheim, Germany) was used and the relative telomerase activity was calculated as the ratio of the absorbance value of the sample to that of the control.

### Statistical analysis

Flow cytometry, stem cell differentiation and telomerase activity measurements were performed on passage three cells. Three experimental replications, both technical and biological were performed. Statistical analysis of the data was carried out using one-way analysis of variance (ANOVA), followed by Tukey's test for multiple comparisons to determine the values that were significantly different (SPSS Version 10, IL, Chicago). Differences were considered statistically significant at p < 0.05.

### Protein extraction and 2D-gel electrophoresis

For analysis of proteome profiles, the cells were grown in defined media (Stem Cell Technologies, USA). Equal number of cells (3x10^5^) was seeded into T-75 flasks in triplicates. When 70% confluency was reached, the cells were washed with ample amount of ice-cold PBS for three times and removed from the plates by scraping. After 10 min centrifugation at 4°C at 2000xg, excess PBS was decanted and 250 μl of cell lysis buffer (2D-rehydration buffer: 8M urea, 2M thiourea, 4% CHAPS, 30 mM Tris pH 8.5, 1x protease inhibitor cocktail) was added onto each cell pellet. To achieve complete lysis, the cells were vigorously vortexed for 1 min in lysis buffer and the supernatant containing the soluble protein fraction was obtained by centrifugation at 20000xg for 30 min at 4°C and stored in Lo-bind tubes (Eppendorf, USA) at -80°C after snap-frozen in liquid nitrogen. Protein concentration was determined by using modified Lowry assay with the BSA standard (BioRad, USA). 80μg of protein was loaded onto immobilized pH gradient strips (IPG) (11cm, pH 5–8) (BioRad, USA) via passive rehydration. Separation based on isoelectric points was performed by using Protean isoelectric focusing cell (BioRad, USA). The strips were run through a stepwise incremental voltage program (250V for 20 min (linear), 4000 V for 2hr (linear) and 10000 V/hr (rapid)). The strips were then subjected to a two-step equilibration in equilibration buffers containing 6M urea, 2% SDS, 0.375M Tris-HCl pH 8.8, 20% glycerol and 2% DTT for the first step and the same buffer without DTT but with iodoacetamide (2.5%) for the second step. Following isoelectric focusing, the strips were subjected to SDS-PAGE using 12% in-house made gels. The gels were stained with Colloidal Coomassie stain (KeraFast, USA) and visualized with VersaDoc4000 MP (BioRad, USA). PDQuest Advance (BioRad, USA) 2DE-analysis software was used for comparison of protein spot profiles. For automated spot detection, parameters used were sensitivity (13.8), spot size scale (3) and minimum peak intensity (258).

### Image analysis and spot cutting

An automated crop tool was used to prepare the images for analysis using PDQuest Advance (BioRad, USA). Automated analyses were performed to detect total spot numbers and volumes within the normalized area. A manual editing tool was used to inspect the determined protein spots detected by the software. Spots that were prone to variation were excluded if they were hard to identify by visual inspection. Spots were cut by using automated spot cutting tool, ExQuest spot cutter (BioRad,USA), and disposed into 96 well plates for protein identification.

### Identification of proteins

Protein identification experiments were performed at Kocaeli University DEKART proteomics laboratory (http://kabiproteomics.kocaeli.edu.tr/) by using ABSCIEX MALDI-TOF/TOF 5800 system. In-gel tryptic digestion of the proteins was performed by using an in-gel digestion kit following the recommended protocol of the manufacturer (Pierce, USA). Zip-Tip cleaning was performed for each digested sample following the recommended protocol (Millipore, USA) before deposition onto a MALDI plate. Peak data were analyzed with MASCOT by using a streamline software, Protein Pilot (ABSCIEX,USA). The parameters for searching were; enzyme of trypsin, 1 missed cleavage, fixed modifications of carbamidomethyl (C), variable modifications of oxidation (M), peptide mass tolerance: 50ppm, fragment mass tolerance: ±0.4 Da, peptide charge of 1+ and monoisotopic. Only significant hits, as defined by the MASCOT probability analysis (*p* < 0.05) were accepted. Classification of the proteins was performed by using a freely available classification system, PANTHER (http://www.pantherdb.org/) [18].

## Results

Adipose tissues were obtained from the abdominal subcutaneous layer of CRF patients with HTBD before and a week after PTX (50 year-old male) and also from a LTBD) patient (70 year-old male) and from a healthy control (40 year-old male). MSCs were isolated from these adipose tissues and characterized to some extent. The stem cells displayed similar morphological properties, which were large, flattened and had fibroblast like shape. No significant morphological changes were detected throughout the study and they displayed similar growth characteristics. Some MSC specific cell surface markers were used to define the cultured cells. The data indicated that the cells expressed some commonly accepted MSCs markers (CD13, CD44, CD90, CD166, CD73, HLA ABC ve CD29) (Table 2 and S1 Fig). These findings were similar to the immunophenotypic MSC characteristics of undifferentiated states of hAT-MSCs reported earlier [19].

**Table 2:** Percentages determined by flow cytometry analysis for markers used to identify hAT-MSCs.

	ADBD LTBD(%)	SHPT HTBD(%)	SHPT After PTX(%)	Control (%)
**CD45**	0.88	0.49	1.07	0.39
**HLA-DR**	0.90	0.10	1.28	0.68
**CD15**	0.63	0.17	1.09	0.29
**CD5**	0.89	0.09	0.98	0.05
**CD4**	0.57	0.09	1.2	0.07
**CD7**	0.68	0.23	1.08	0.03
**CD14**	1.17	0.27	1.92	1.36
**CD17**	1.81	0.49	2.02	1.95
**CD11b**	2.2	0.83	1.75	1.74
**CD13**	97.98	76.6	81.17	72.96
**CD34**	6.8	4.45	0.04	3.92
**CD106**	1.40	0.15	1.06	0.04
**CD44**	99.49	59.08	94.94	75.73
**CD90**	92.33	74.6	71.82	77.60
**CD166**	89.37	73.1	43.92	76.48
**CD10**	0.12	0.93	0.03	0.08
**CD8**	0	0.35	0	0.06
**CD33**	0.27	2.0	0.03	0.05
**CD71**	1.66	20.2	2.07	0.07
**CD146**	47.77	1.96	55.62	1.95
**CD73**	99.52	70.92	99.21	79.52
**HLA-ABC**	87.90	49.03	81.01	77.17
**CD29**	99.53	74.72	99.63	80.67
**CD123**	0.07	1.76	0.22	0.01
**CD138**	0.01	5.75	0.46	0.04
**CD19**	1.74	0	0.67	0.28
**CD3**	1.46	0	0.78	0.4
**HLA- G**	2.33	0.17	0.69	7.15
**CD133**	0.06	0	0.65	0

**Fig 1:**
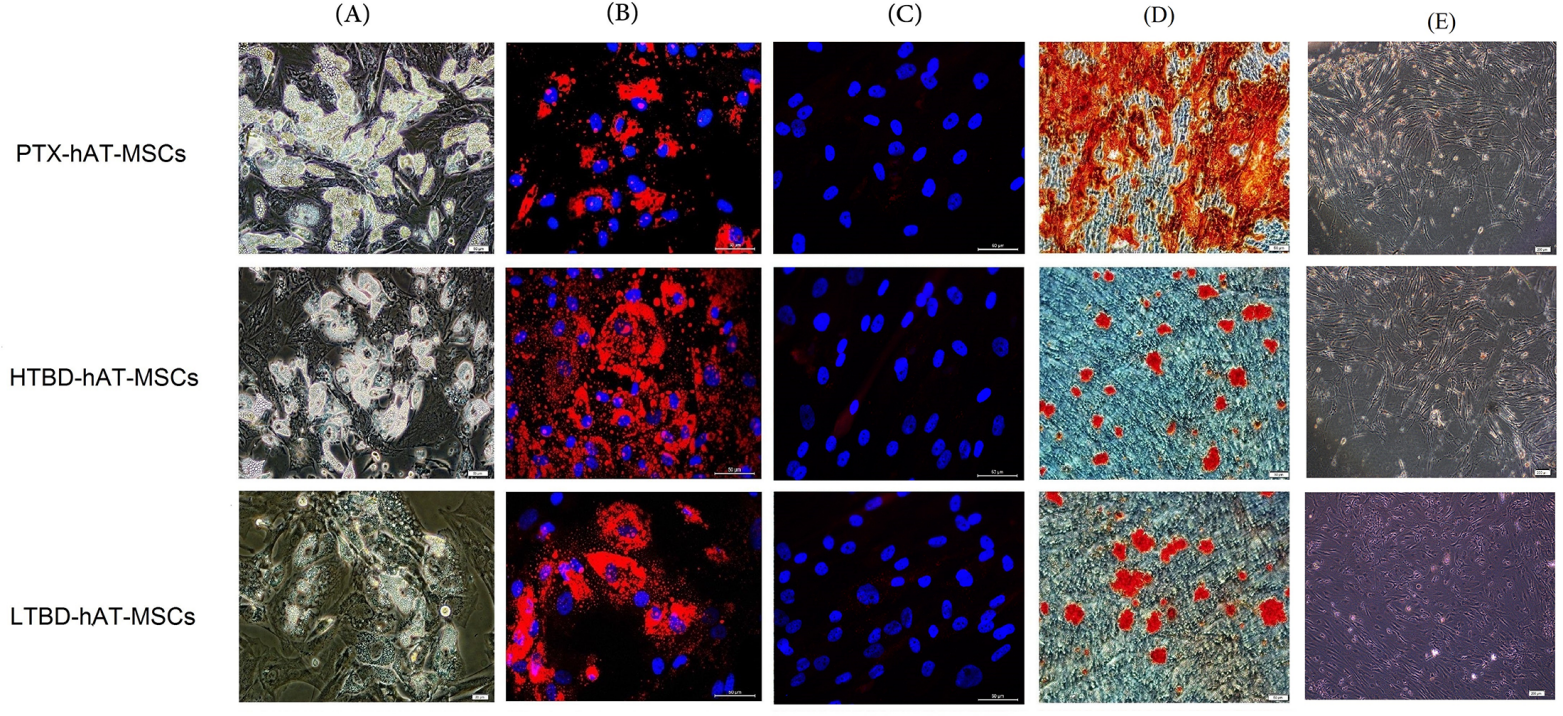
Differentiation of hAT-MSCs from PTX-hAT-MSCs, HTBD-hAT-MSCs and LTBD-hAT-MSCs. The cells were subjected to (A and B) adipogenic and (C) osteogenic differentiations as described in material and methods section. (A) Inverted microscope image of the cells subjected to adipogenic differentiation. (B) Oil Red-O staining of adipogenic cells differentiated from hAT-MSCs. (C) Alizarin Red staining of osteogenic cells differentiated from hAT-MSCs.

To assess multipotential characteristics of the stem cells, measurement of differentiation potential is required. Each cell line was subjected to adipogenic and osteoblastic differentiation and differentiations were characterized by using immunohistochemical and fluorescence techniques. At the beginning of adipogenic differentiation, the presence of lipid droplets was evident and towards the end of differentiation, the lipid droplets enlarged and invaded the entire cytoplasm (Fig 1A). During osteoblastic differentiation, cellular aggregates were observed in the culture plates and were gradually increased until the end of the experiment. These aggregates were characterized by the presence of amorphous material deposits (Fig 1B). Alizarin red S stained these nodular aggregates in osteoblastic cultures, demonstrating that the amorphous deposits observed under the microscope were in fact calcium deposits (Fig 1C). At day 30, positive alizarin red aggregates were larger and stained intensively indicating the presence of a more extensive calcium deposition. Control cultures showed only minimal background staining.

**Fig 2:**
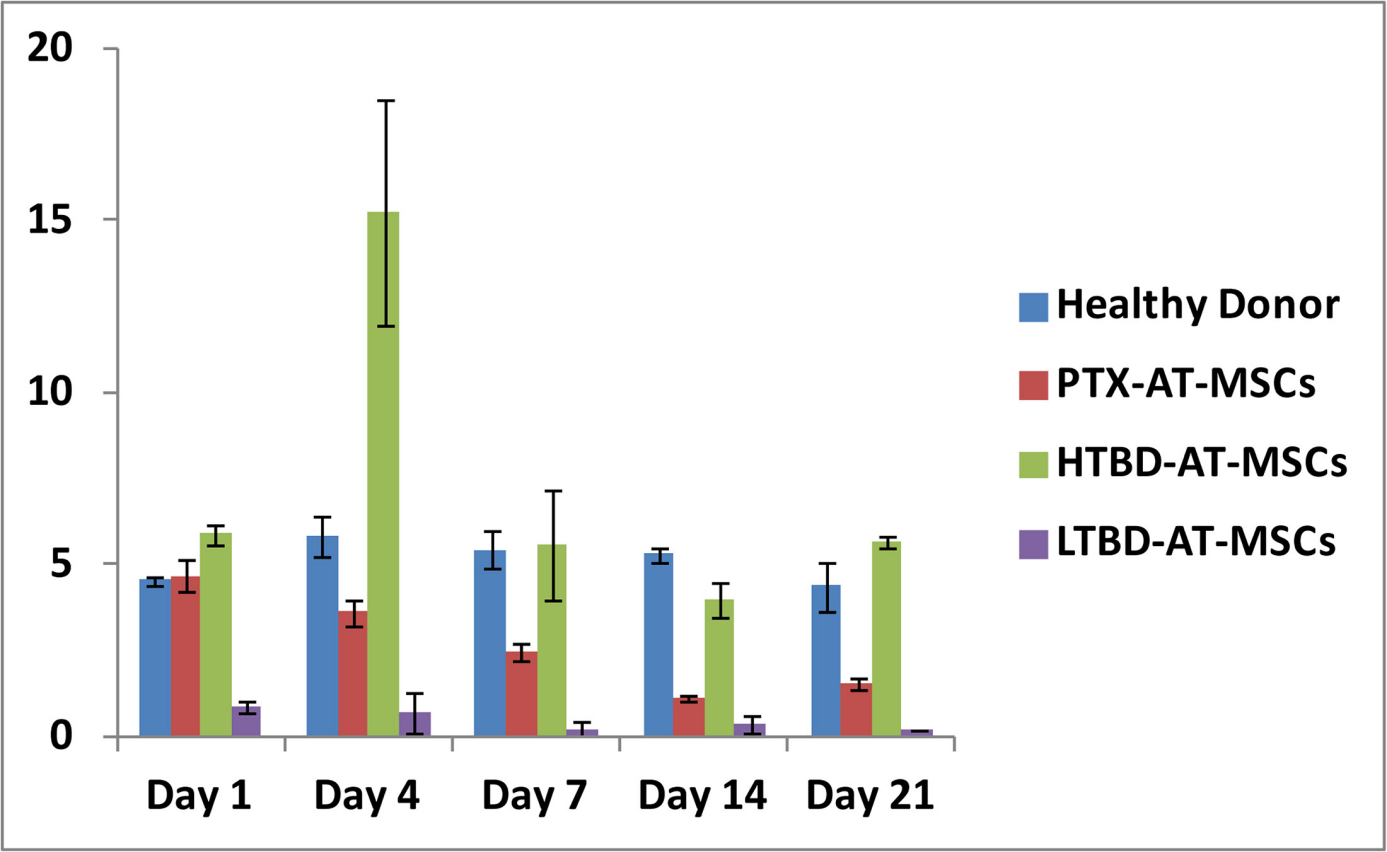
Alkaline phosphatase activity of hAT-MSCs. PTX: Stem cells isolated from human adipose tissue after parathyroidectomy operation. HTBD: Stem cells isolated from human adipose tissue obtained from a patient with secondary hyperparathyroidism (High turnover bone disease). LTBD: Stem cells isolated from human adipose tissue obtained from a patient with adynamic bone disease (Low turnover bone disease).

ALP activity, another osteoblastic differentiation marker, gradually decreased in all cell lines except HTBD-hAT-MSCs, which reached to its peak activity at day 4 (Fig 2). In overall, the ALP activity was higher in cell cultures generated from the stem cells isolated from the patient tissue with HTBD than from the stem cells of the same patient after PTX operation (PTX-hAT-MSCs). ALP activity in the cell cultures generated from the stem cells isolated from the patient with LTBD was almost undetectable.

**Fig 3:**
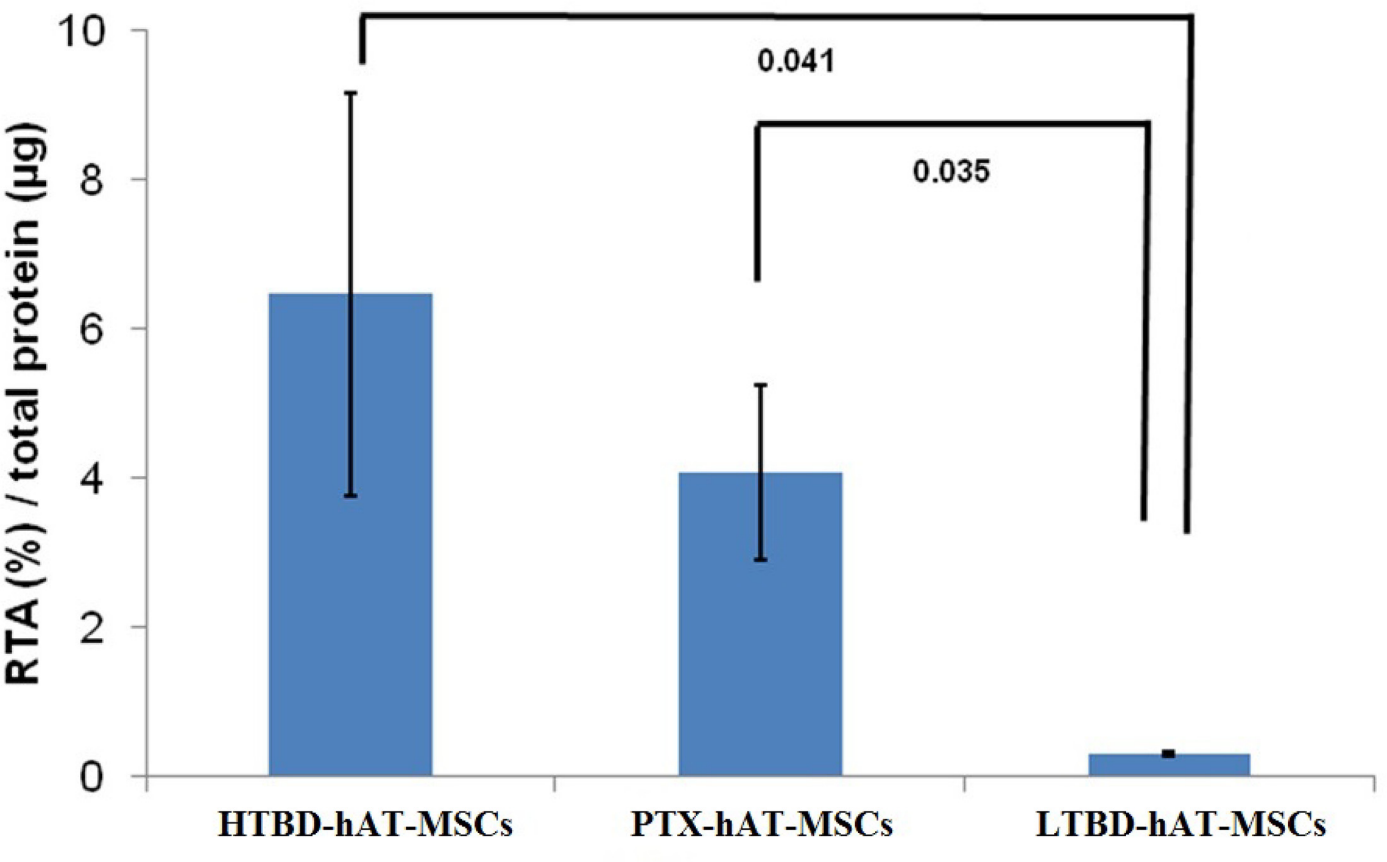
Telomerase activities of hAT-MSCs obtained from HTBD-hAT-MSCs, PTX-hAT-MSCs and LTBD-hAT-MSCs. HTBD: Stem cells isolated from human adipose tissue obtained from a patient with secondary hyperparathyroidism (High turnover bone disease). LTBD: Stem cells isolated from human adipose tissue obtained from a patient with adynamic bone disease (Low turnover bone disease). The activities were measured by conventional telomeric repeat amplification protocol (TRAP) and relative telomerase activities (RTA) were calculated with respect to the control template equivalent to 0.001 mol/*μ*L DNA.

Telomere shortening is an indicator of stem cell aging and can be monitored by measuring the telomerase activity. Telomerase activities of PTX-hAT-MSCs, HTBD-hAT-MSCs and LTBD-hAT-MSCs were measured at P3. The telomerase activity of HTBD-hAT-MSCs was 6.47±2.69 amol/μg total protein, PTX-hAT-MSCs was 4.09±1.18 amol/μg total protein and LTBD-hAT-MSCs was 0.03±0.02 amol/μg total protein (Fig 3).

**Fig 4:**
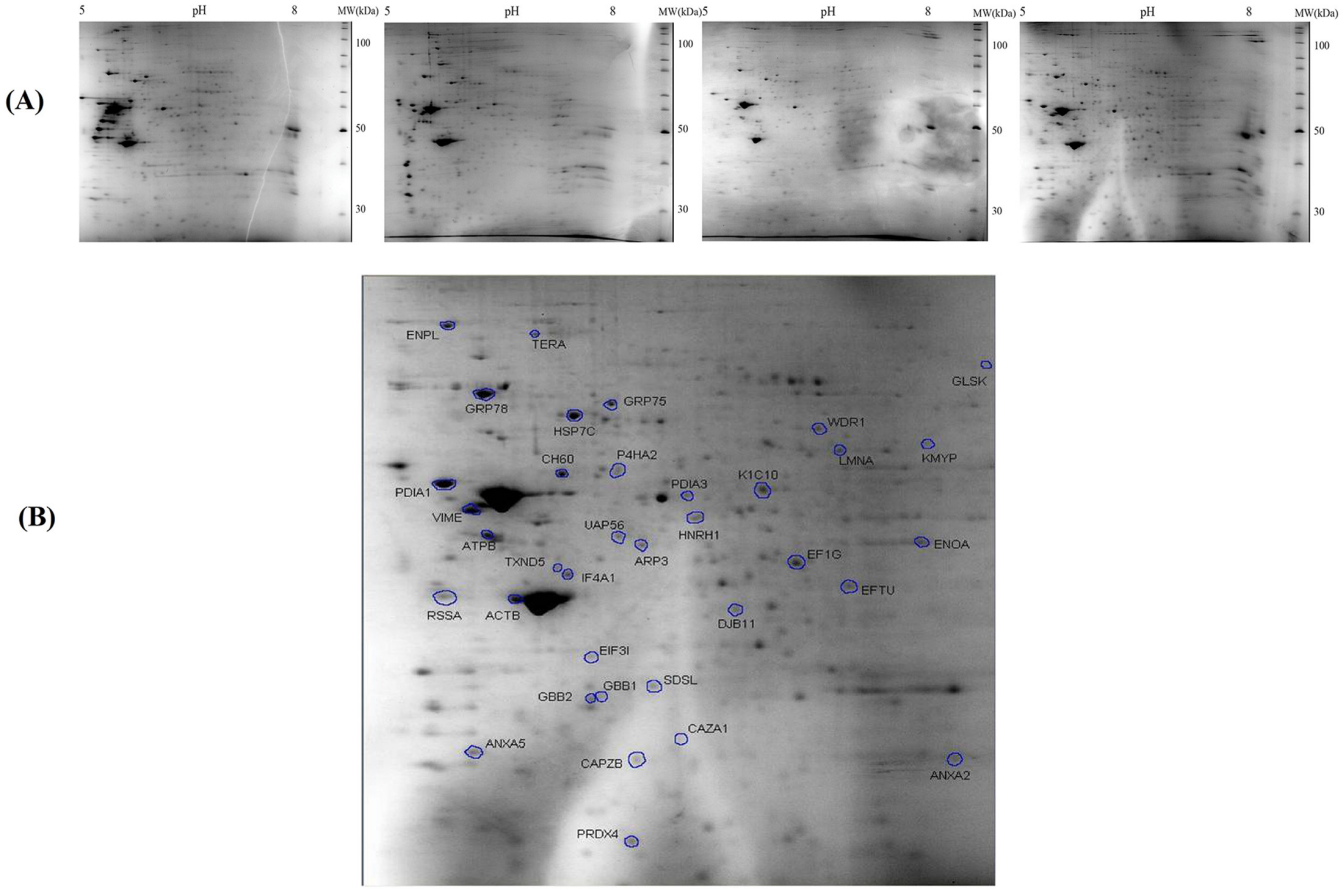
Comparative proteome analysis of hAT-MSCs obtained from HTBD-hAT-MSCs, PTX-hAT-MSCs and LTBD-hAT-MSCs. HTBD: Stem cells isolated from human adipose tissue obtained from a patient with secondary hyperparathyroidism (High turnover bone disease). LTBD: Stem cells isolated from human adipose tissue obtained from a patient with adynamic bone disease (Low turnover bone disease). (A) MSCs were subjected to protein isolation and proteins were loaded onto pH 5 to pH 8 IPG strips for the first dimension and precast SDS-PAGE gels for the second dimension separation and stained with Colloidal Coomassie Blue for 24 hr after 24 hours of fixation. The gels are representative of three gels from each MSC type. (B) The 2DE gel showing the protein spots that were cut from the gels and were subjected to MALDI-TOF/TOF analysis for identification.

**Fig 5:**
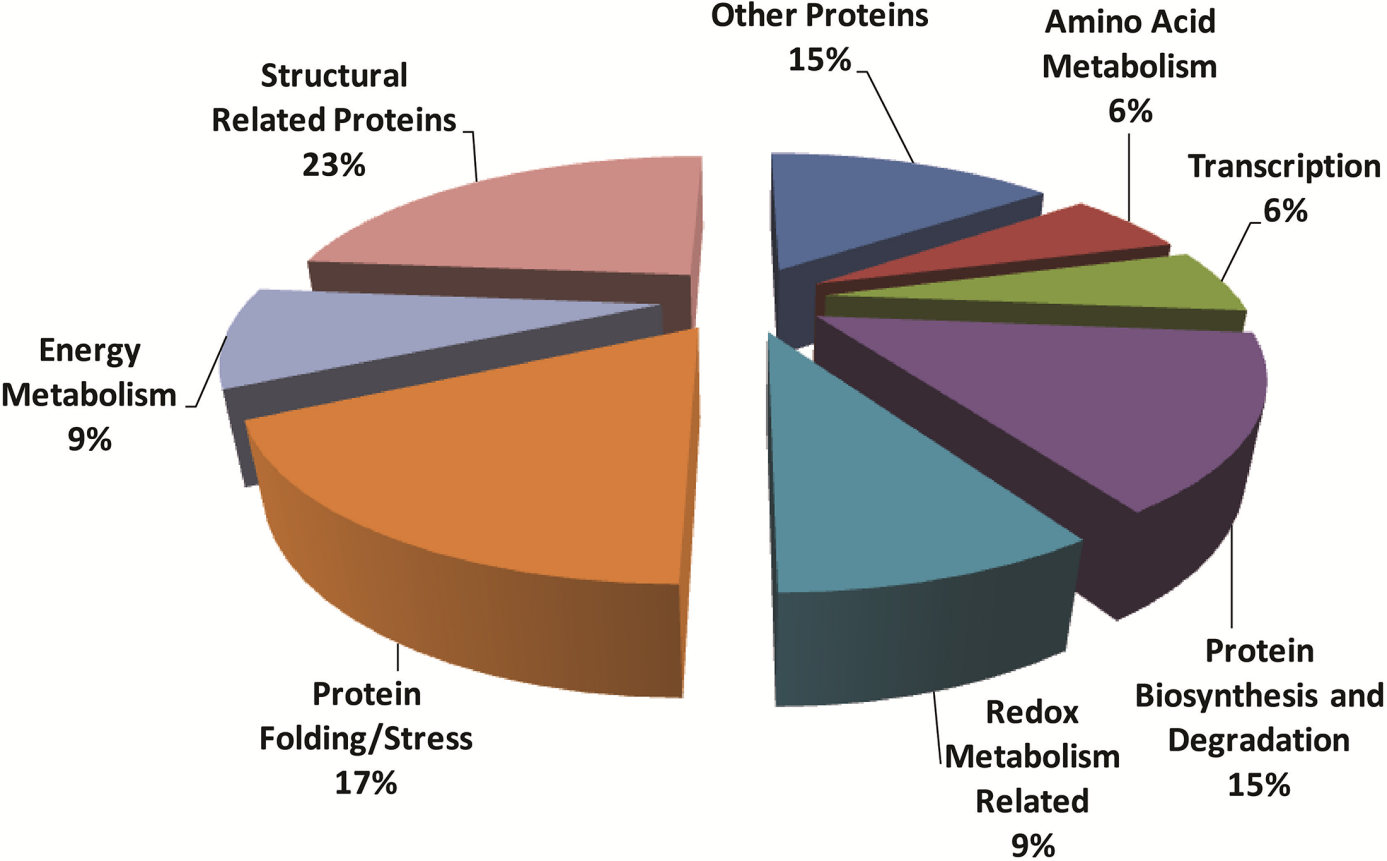
Classification of identified proteins based on their molecular function and their involvement in biological processes. Pie chart representing the distribution of the identified proteins based on their molecular function and biological processes. Assignments were made on the basis of information from PANTHER analysis (http://www.pantherdb.org/) as well as NCBI (http://www.ncbi.nlm.nih.gov/pubmed) and Swiss-Prot/TrEMBL annotations (http://www.expasy.org/).

To understand the complexity observed in these cells at the molecular level, a comparative 2DE-based proteomic study was performed. Well-resolved and reproducible 2DE gel maps were produced as shown in Fig 4. An average of 410±30 spots per analytical gel was detected when the gels were subjected to automated spot detection and analysis. However, the number of spots that reliably matched to every member was 71 with an overall mean coefficient of 89.55 percent. The rest of the spots were either not matched or were not expressed by all three stem cell lines. By using PDQuest advance gel analysis software, changes in spot intensities among these 71 matching spots were compared. Spots that were up or down regulated more than 2-fold was considered to be subject to regulation. Ratios for up and down regulated protein spots were given in Table 3. To identify the conserved protein spots, the spots were cut from a preparative gel with an automated spot cutting instrument and subjected to in-gel tryptic digestion followed by MALDI-TOF/TOF analysis. A total of 32 protein spots were identified. The identified proteins were subjected analysis based on their molecular function and their involvement in biological processes (Fig 5). Proteins were classified into seven distinct groups, namely (1) structural proteins, (2) proteins involved in protein folding and stress, (3) redox metabolism, (4) protein biosynthesis and degradation, (5) proteins involved in transcription, (6) energy and (7) amino acid metabolisms. There were five proteins which could not be classified with the others and were evaluated separately.

**Table 3:** Proteins identified by MALDI-TOF/TOF analysis and classification according to their function. The last three columns represent the values for the regulation rations of the proteins in comparison to the control.

Function of the proteins	Gene Name-Protein name	HTBD/ control	After PTX OP/control	LTBD/control
**Structural**	VIM -Vimentin	0,03	0,06	0,05
	ACTG1-Actin Cytoplasmic 1	0	0	0
	CAPZB -F-acting-capping protein subunit beta-alfa-1	28491	0,12	0,56
	WDR1-WD repeat-containing protein 1	0,59	0	0,43
	LMNA -Prelamin A/C	0,4	0	0,29
	KRT1 -Keratin,type1 cytoskeleton 10	18994	0	0,48
	ACTR3 -Actin related protein	43101	0,04	41883
**Protein folding/stress**	DNAJB11-DnaJ homolog subfamily B member 11	0,67	0	0,42
	HSPA5-78kDa glucose-regulated protein	0,3	0,47	0,3
	HSP90B1-Endoplasmin	42005	41852	0,77
	HSPA9-Stress-70 protein, mitochondrial	0,5	0,99	0,55
	HSPD1-60kDa heat shock protein, mitochondrial	0,25	0,18	0,35
	HSPA8-Heat shock cognate 71kDa protein	0,28	0,48	0,41
**Redox metabolism**	PDIA4-Protein disulphide-isomerase A	0,52	0,01	0,75
	PRDX6-Peroxiredoxin	34335	0	0,27
	TXNDC5-Thioredoxin domain-containing protein 5	0	0	4,36
**Protein biosynthesis/degradation**	EIF4AI-Eukaryotic initiation factor 4A-I	0,19	0	0,26
	TUFM-Elongation factor TU	0,41	0	0,21
	EIF3I-Eukaryotic translation initiation factor 3 subunit I	0,55	0	0
	EEF1G-Elongation factor I gamma	41760	0,02	41699
	RPSA-40S ribosomal protein SA	42736	33604	0,38
**Transcription**	DDX39B-RNA helicase DDX39B	0,82	0	0,44
	HNRNPH1-Heterogeneous nuclear ribonucleoprotein H	14246	0,07	0,73
**Energy metabolism**	ENO1-Alpha enolase	0,98	0,72	0,37
	ATP5B-ATP synthase subunit beta, mitochondrial	0,62	0,83	0,6
	PKM -Pyruvate kinase	14,6	18,1	44,27
**Amino acid metabolism**	SDS-Serine dehydratase	16438	0	0,71
	P4HA2-Prolyl 4-hydroxylase subunit alpha-2	0,24	0,01	41671
**Others**	VCP-Transitional endoplasmic reticulum ATPase	0,8	0,02	0,75
	ANXA5-Annexin A5	41883	17899	41699
	GLS-Glutaminase kidney isoform, mitochondria	18994	0	0,48
	ANXA2-Annexin A2	0,26	0,48	0,1
	GNB2-Guanine nucleotide-binding protein G(I)/G(S)/G(T)subunit beta-2	42036	0,66	41275

## Discussion

There is a possibility that stem cells will be used to cure some of the incurable human diseases. However, for this to happen in the near future, novel stem cell therapies which rely upon an understanding of the biology of these interesting cells have to be developed. Stem cells exist in a specialized environment in the body known as “the niche”. The niche is known to control the behavior of the stem cells and ensures their survival. Most human stem cell niches are deep in the body and so difficult to study. Therefore, the question of how changes in stem cell niches of the host affect the stem cell properties is among the most challenging questions to be answered [20, 21]. One approach to answer this type of question is perhaps to isolate stem cells from the same niche of the same individual under two different conditions for comparison such as before and after the treatment of a disease that may affect both the niche and the stem cell properties. In this study, the stem cells from an easily accessible stem cell niche, subcutaneous adipose tissue, were isolated from an individual with CRF with HTBD who underwent PTX operation were used. This allowed us to compare the same type of stem cells isolated from the same type of niche under two different host conditions to answer the question of how changes in the stem cell environment of the host affected stem cell properties. Furthermore, we studied stem cells from an aged-match healthy individual as the control and the stem cells from a patient with CRF accompanied by LTBD. Comparisons were made among the stem cells by measuring ALP activities, telomerase activities, adipogenic and osteoblastic differentiation potentials and changes at the proteome level.

### Comparison of ALP activities revealed the presence of predefined molecular mechanisms in stem cells

ALP activity is known to reflect the rate of bone turnover in adult individuals [22]. However, to the best of our knowledge, in most studies, although ALP activity during the osteoblastic differentiation was measured in MSCs, the results were not evaluated in terms of their relevance to the rate of bone turnover. In this study, hAT-MSCs of the patient with HTBD displayed significantly higher ALP activity during osteoblastic differentiation, then the hAT-MSCs of the same patient after PTX operation. In addition, hAT-MSCs of the CRF patient with LTBD had the lowest ALP activity (Fig 2). These observations implied that ALP activity of hAT-MSCs during osteoblastic differentiation may be correlated with the bone turnover status. The stem cells should be expected not to have predefined molecular mechanisms leading them to differentiation. However, our findings indicated and supported, that there are predefined molecular mechanisms to underline the features of the observed host diseases in stem cells.

### Telomerase activity in stem cells was not affected by bone turnover status of the host

Age-related telomerase shortening due to loss of telomerase activity in AT-MSCs might be an indicator of host aging from whom the stem cells were obtained [23]. We observed a donor-age associated decrease in telomerase activities. The least telomerase activity was observed in the elderly patient with LTBD (Fig 3). The patient who underwent PTX displayed low bone turnover in a week after the operation, although the stem cells isolated from this patient after PTX still had relatively high telomerase activity indicating that the turnover status of the bone might not really affect the level of telomerase activity.

### Changes in the host status cause changes in global protein expression profile in the hAT-MSCs

Most of the identified proteins were classified into seven categories (Table 2 and Fig 5). Some of these play critical roles in cellular architecture. Also, proteins that were part of the protein folding machinery were described. Some of those were inducible chaperons that were expressed under stress conditions. Transcription, protein biosynthesis and degradation related proteins that we detected indicated the presence of active cell growth. Redox metabolism-related scavenger proteins that were detected may be an indicator of active cell growth as well. The presence of apoptosis, transcription, protein biosynthesis/degradation and energy metabolism-related proteins indicated cellular self-renewal and proliferation. Some of the proteins identified in this study were similar to the proteins reported by the earlier studies [24, 25].

### The abundant proteins present in the high turnover state, but absent in the low turnover state require attention

There were two structural proteins that required our attention since their abundances were high before PTX in comparison to the control. These proteins were F-acting-capping protein and keratin, type 1cytosceleton 10. These two proteins were not regulated in AT-MSCs of the patient with LTBD in comparison to the control, although there was a high level of regulation in AT-MSCs of the patient with HTBD in comparison to the control. This finding indicated that these proteins may play pivotal roles in determining the status of the bone turnover. This interpretation might make sense since the actin cytoskeleton controls cell shape, mobility, division and intracellular transports. Although the functions of these two proteins in stem cells are not well established, their roles in cancer cells are known in detail [26]. They are involved in cell growth, proliferation, stiffness, movement of invasiveness in cancer cells [27]. The F-acting capping protein which is an actin regulator and constructed as a heterodimer consists of alpha and beta subunits and functions as a tumor suppressor protein. F-actin-capping protein subunit β plays a role for actin binding and regulates cell morphology and cytoskeleton organization under normal circumstances [26–28].

There is no study establishing a relationship between Keratin type 1 cytoskeleton 10 protein and bone turnover. However, the protein content of blood in 25 patients with bone fracture was reported [29]. Twelve of the 213 identified proteins were found to be related to the bone and cartilage metabolism. Keratin, type 1cytoskeloton 10 were present among them, and it was claimed that this protein should have a connection with the bone formation [29]. Our study is the first study showing a change in expression of keratin type 1 cytoskeleton 10 protein in response to changes in bone turnover. However,a study reported a correlation between organization of MSCs cytoskeleton elements and, changes in cell morphology and expression of some markers [30]. In that study, the researchers found out that cell shape of MSCs and their cytoskeleton organization have changed during osteoblastic differentiation [30]. This reorganization of the cytoskeleton may occur as an early response to changes in some hormones such as dexamethosone, growth hormone, parathyroid hormone. This early report supported our findings about changing the expression of some structural proteins in response to bone turnover, which is affected by the PTH level [31].

Redox regulation is an essential physiological process in the survival of all cell types [32]. Imbalance in a redox system creates oxidative stress in the cells, resulting in an impairment of cellular function, lipid peroxidation, protein degradation and nucleic acid breakage. There was a high level of peroxiredoxin expression in AT-MSCs of the patient with HTBD but not after the PTX operation and not with the patients with LTBD.

Peroxiredoxin is proposed to play a role in cell signaling by regulating radical oxygen species [33]. In general, the increase in peroxiredoxin levels can be interpreted as an indicator of active cell growth, which produces radical oxygen species (ROS). However, there is a report about the relationship between osteoclastogenesis and peroxiredoxin-2 through the pathway induced by RANKL (receptor activator of NF-κB ligand), which is a well known osteoclastic biomarker. RANKL induces multiple signaling pathways causing long lasting ROS increase and Ca^2+^ oscillations in bone morrow-derived monocytes, which result in differentiation into osteoclasts [34]. The physiological consequence of RANKL signaling activation pathway is imitated in peroxiredoxin-2 knocked out mice and the results demonstrated a clear relationship among ROS, ROS scavenger proteins and the decrease in bone density. This study indirectly support our findings about the relation between the increases in peroxiredoxin levels and HTBD. In addition, there is a report about the usefulness of peroxiredoxin-2 as a biomarker to predict chemotherapy response in osteosarcome treatment, although the molecular mechanism behind the increase in peroxiredoxin-2 levels in osteosarcomes with poor response is not explained [35].

Heterogeneous nuclear ribonucleoproteins (hnRNPs) constitute a set of polypeptides that bind heterogeneous nuclear RNA, the transcripts produced by RNA polymerase II [36]. There is no report about the effect of hnRNP expression on bone turnover. However, hnRNP is a multifunctional protein that is involved not only in processing heterogeneous nuclear RNAs (hnRNAs) into mature mRNAs, but also acting as *trans*-factors in regulating gene expression [37]. It is our prediction that the change in abundance of hnRNP might be the consequence of its trans-acting function.

Two proteins were identified in stem cells isolated from high bone turnover state related to the metabolic acidosis. This is a relevant finding because the patient we studied suffered from CRF and chronic metabolic acidosis. The first one was serine dehydratase protein (SDH), which catalyzes pyridoxal phosphate (PLP) dependent deamination of serine and threonine to produce pyruvate and α-ketobutyrate, respectively. This is one of the few enzymes that directly release ammonia from amino acids. This enzyme is reported to play an important role in adaptation to metabolic acidosis [38].

Glutaminase (GA) kidney isoform-1 is a well studied enzyme with well-defined catalytic properties. This enzyme is known to catalyze the reaction that produces ammonium from glutamine (GLN) in renal tubular cells; so it plays an important role in maintaining acid-base homeostasis [39]. Surprisingly, in this study, GA was also detected in AT-MSCs of the patient with SHPT (HTBD) before the PTX operation. GA has three different isoforms: GA Isoforms 1, 2 and 3. Isoform 1 is highly expressed in the brain and the kidney. There is no report stating expression of GA isoform-1, 2 or 3 in adipose tissue. However, since the stem cells isolated from the hAT displayed a higher abundance of this enzyme, the enzyme should either be expressed in AT at a low level or the AT has the potential to express GA isoform 1, when it is necessary. The reason why we observed such a high level GA isoform-1 expression in stem cells in HTBD patient might be due the fact that hyperparathyroidism is a possible cause for metabolic acidosis [40].

## Conclusion

This is the first study providing proteomic details of hAT-MSCs isolated from the same host under different conditions. Some of the identified and regulated proteins may be linked to the disease status of the host. However, some proteins that were identified did not reveal any association between the host’s disease status and the metabolic changes of the stem cells. We are aware of the fact that the study was undertaken *in vitro* and changes that reflect *in vivo* conditions may be missing or may not reflect the reality. However, any observation *in vitro* is worthy of investigation in *in vivo* and may provide valuable clues about the metabolic events. The list of the candidate proteins generated in this study needs further attention to establish firm, conclusive connections between disease-associated changes in stem cell metabolism.

## Supporting Information

S1 Fig Flow cytometry analysis of of hAT-MSCs obtained from HTBD-hAT-MSCs, PTX-hAT-MSCs, LTBD-hAT-MSCs and the control. HTBD: Stem cells isolated from human adipose tissue obtained from a patient with secondary hyperparathyroidism (High turnover bone disease). LTBD: Stem cells isolated from human adipose tissue obtained from a patient with adynamic bone disease (Low turnover bone disease).
(TIF)
